# Effects of Ozone on Sickness and Depressive-like Behavioral and Biochemical Phenotypes and Their Regulation by Serum Amyloid A in Mice

**DOI:** 10.3390/ijms24021612

**Published:** 2023-01-13

**Authors:** Kristen K. Baumann, W. Sandy Liang, Daniel V. Quaranta, Miranda L. Wilson, Helina S. Asrat, Jarl A. Thysell, Angelo V. Sarchi, William A. Banks, Michelle A. Erickson

**Affiliations:** 1 Geriatric Research Education and Clinical Center, VA Puget Sound Healthcare System, Seattle, WA 98108, USA; 2 Division of Gerontology and Geriatric Medicine, Department of Medicine, University of Washington, Seattle, WA 98195, USA

**Keywords:** ozone, inflammation, depression, serum amyloid A, kynurenine

## Abstract

Ozone (O_3_) is an air pollutant that primarily damages the lungs, but growing evidence supports the idea that O_3_ also harms the brain; acute exposure to O_3_ has been linked to central nervous system (CNS) symptoms such as depressed mood and sickness behaviors. However, the mechanisms by which O_3_ inhalation causes neurobehavioral changes are limited. One hypothesis is that factors in the circulation bridge communication between the lungs and brain following O_3_ exposure. In this study, our goals were to characterize neurobehavioral endpoints of O_3_ exposure as they relate to markers of systemic and pulmonary inflammation, with a particular focus on serum amyloid A (SAA) and kynurenine as candidate mediators of O_3_ behavioral effects. We evaluated O_3_-induced dose-, time- and sex-dependent changes in pulmonary inflammation, circulating SAA and kynurenine and its metabolic enzymes, and sickness and depressive-like behaviors in Balb/c and CD-1 mice. We found that 3 parts per million (ppm) O_3_, but not 2 or 1 ppm O_3_, increased circulating SAA and lung inflammation, which were resolved by 48 h and was worse in females. We also found that indoleamine 2,3-dioxygenase (*Ido1*) mRNA expression was increased in the brain and spleen 24 h after 3 ppm O_3_ and that kynurenine was increased in blood. Sickness and depressive-like behaviors were observed at all O_3_ doses (1–3 ppm), suggesting that behavioral responses to O_3_ can occur independently of increased SAA or neutrophils in the lungs. Using SAA knockout mice, we found that SAA did not contribute to O_3_-induced pulmonary damage or inflammation, systemic increases in kynurenine post-O_3_, or depressive-like behavior but did contribute to weight loss. Together, these findings indicate that acute O_3_ exposure induces transient symptoms of sickness and depressive-like behaviors that may occur in the presence or absence of overt pulmonary neutrophilia and systemic increases of SAA. SAA does not appear to contribute to pulmonary inflammation induced by O_3_, although it may contribute to other aspects of sickness behavior, as reflected by a modest effect on weight loss.

## 1. Introduction

O_3_ is a widespread toxicant in air pollution that is harmful to human health. Epidemiological studies have shown that short-term increases in ambient O_3_ are associated with increased mortality and morbidity [1,2,3]. Healthy humans acutely exposed to O_3_ experience temporary adverse effects, which include decreased pulmonary function and increased pulmonary inflammation [4,5,6]. Although the lungs are the primary target organ for direct oxidant activities of O_3_, growing evidence supports the idea that O_3_ exposure may affect distal organs, including the brain [7,8]. Increased oxidative stress, neuroinflammation, blood-brain barrier (BBB) disruption, and neurotransmitter dysfunction have been associated with O_3_ exposure in rodents and humans [9,10,11,12,13]. Epidemiological studies also implicate O_3_ as a possible risk factor for neurological conditions such as cognitive impairment/decline [14,15,16], Alzheimer’s disease (AD) [17], and depression [18,19,20,21] in certain populations. Acute exposure to O_3_ can elicit neurological symptoms such as headaches, dizziness, fatigue, and mental tension in humans [22,23], suggesting that the CNS effects of O_3_ may contribute to exposure-associated morbidities such as feelings of illness in the short term. However, the mechanisms by which O_3_ elicits its effects on the CNS are largely unclear since the direct oxidant activities of O_3_ are limited to the lungs.

One emerging hypothesis on how O_3_ affects the CNS is through a lung-brain axis mechanism [12], whereby initial O_3_-induced pulmonary damage and local inflammation elicit a systemic inflammatory response and the release of circulating factors that can affect CNS functions. Often, neuroimmune communication pathways involve the systemic release of pro-inflammatory cytokines and chemokines [24], but blood and brain levels of pro-inflammatory cytokines and chemokines are not increased in mice exposed to O_3_ [12,25]. However, other inflammation-associated factors, such as kynurenine and SAA, are increased in the blood of O_3_-exposed rats and mice, respectively [25,26]. Kynurenine is a tryptophan metabolite that is elevated in the blood and brain in response to inflammation through the activation of its rate-limiting enzyme, indoleamine 2,3-dioxygenase (IDO) [27]. The activation of IDO has been shown to mediate depressive-like behaviors following inflammatory stimuli [28,29], and kynurenine elevations have been implicated in depression [30] and in other neurological diseases [31]. Kynurenine can cross the intact BBB via the large neutral amino acid transporter, LAT-1, and it has been estimated that most of the kynurenine in brain is derived from the circulation [32]. SAA is an acute phase protein and two of its isoforms, SAA1 and SAA2, are produced mainly by hepatocytes, are markedly upregulated in response to inflammatory insults, and circulate primarily as high density lipoprotein (HDL) [33]. We recently showed that SAA1 and SAA2 can cross the intact mouse BBB and that SAA1/2 concentrations in brain, liver, and blood increase following an acute O_3_ exposure in mice [25]. In the same study, SAA levels in the blood correlated significantly with O_3_-induced pulmonary inflammation. Elevations of SAA in blood have also been associated with pulmonary inflammation during acute exacerbations of COPD [34], suggesting that pulmonary inflammation is coupled with SAA production by the liver in human disease. 

The current evidence that both SAA and kynurenine are induced by O_3_, are derived in part from peripheral sources, and can cross the BBB, contribute to depressive-like behaviors in mice [29,35], and are associated with depressive symptoms in humans [30,36] suggests that they could be mediators of neurobehavioral changes following O_3_ exposure. Therefore, we sought to further characterize the O_3_-induced SAA and kynurenine increases and their relation to pulmonary inflammation and acute changes in behaviors. Behaviors associated with sickness and depression can be overlapping and include malaise, anorexia, reduced locomotor activity, disinterest in social interactions, lethargy, reduced grooming, weight loss, anxiety, anhedonia, and memory impairment [37,38]. Sickness behaviors are distinguished from depression, in part, by their manifestation as an adaptive response to infection or injury [38]; however, inflammatory challenges that initially cause sickness behaviors can also lead to depressive symptoms. For example, pharmacological treatment with interferon-α (IFN-α) can precipitate a major depressive episode in 15–40% of patients [39]. In humans experimentally treated with a low dose of bacterial lipopolysaccharide (LPS), symptoms of anxiety, depressed mood, and memory impairment are transiently induced and are more severe in women [40,41]. In mice, a single low-dose injection of LPS can contribute to both sickness and depressive-like behaviors, which are expressed in distinct temporal patterns [28,30]. Behaviors such as a reduced sucrose preference and increased immobility in the tail suspension test persisted after other behaviors associated with cytokine-induced sickness responses, including reduced food intake and locomotor activity, resolved [28]. To our knowledge, the temporal patterns of sickness and depressive-like behaviors as they relate to inflammatory changes following acute O_3_ exposure have not been evaluated in mice. However, such information would be important for the design of studies that evaluate depressive-like behaviors, cognition, or other behavioral phenotypes that could be influenced by acute behavioral responses related to sickness. 

**Figure 1:**
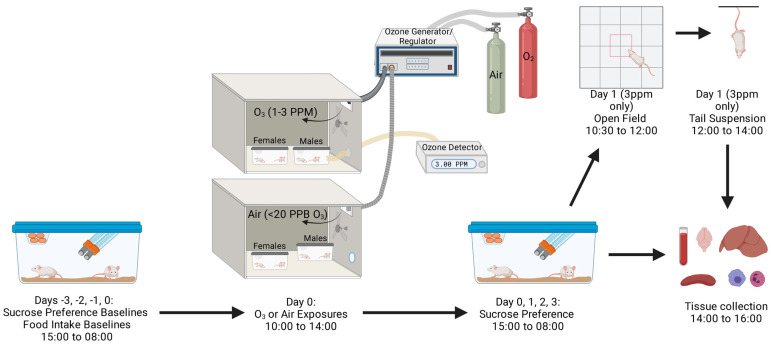
Schematic for the schedule of O_3_ exposures, behavior testing, and tissue collection. Created with BioRender.com.

The first goal of our study was to evaluate the dose- and time-responses of sickness and depressive-like behavioral changes in female Balb/c and CD-1 mice, with respect to pulmonary inflammation and damage and SAA levels in blood. We also aimed to determine whether sex influences any of our measured parameters. A schematic of our experimental design is shown in Figure 1. We found that there is a defined O_3_ dose-threshold and time-window that induces elevations in SAA, pulmonary inflammation, and sickness and depressive-like behaviors, and there are sex differences in these responses to O_3_. We further showed that tissue *Ido1* mRNA expression and kynurenine levels in blood were significantly altered by O_3_ and that O_3_ induced similar biochemical and behavioral responses in female Balb/c and CD-1 mice, as well as in C57BL/6J mice, highlighting the robustness of the response across mouse strains. Because SAA in blood strongly correlates with pulmonary inflammation [25], our second goal was to determine whether SAA contributes to lung inflammation and serum kynurenine elevations, as well as sickness and depressive-like behaviors following O_3_ exposure using SAA knockout mice.

## 2. Results

### 2.1. Dose Effects of O_3_ on Body Weight, Serum SAA, and Pulmonary Damage and Inflammation

**Figure 2:**
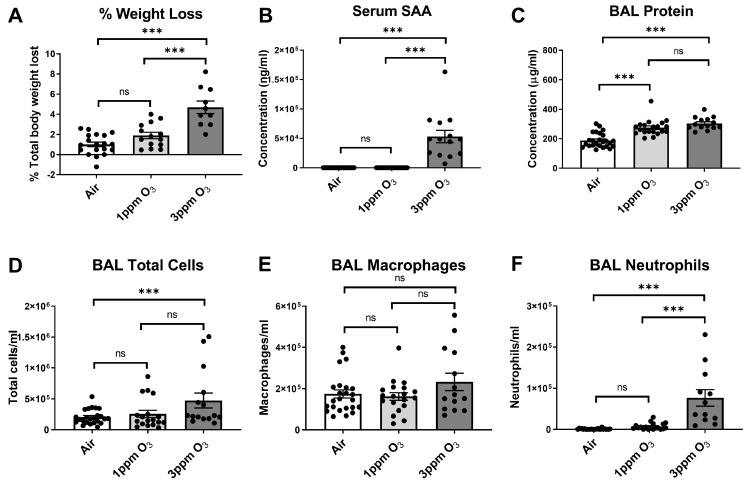
Effects of O_3_ concentration on weight loss (**A**), serum SAA (**B**), BAL total protein (**C**), and cellular markers of acute pulmonary inflammation in female Balb/c mice (**D**–**F**). All mice were studied 24 h post-exposure. The means ± SEM are graphed. N = 10–24/group, *** *p* < 0.001 vs. indicated groups, ns = not significant (*p* > 0.1 except when reported).

Our prior work demonstrated that female Balb/c mice exposed to 3 ppm O_3_ had significantly increased levels of SAA protein in blood and brain 24 h after exposure [25]. We first wanted to determine whether SAA induction also occurred at lower concentrations of O_3_ and chose 1 ppm as a lower dose because it has been used by others to evaluate CNS endpoints following acute exposure to O_3_ [12,13]. We also evaluated weight loss and markers of pulmonary vascular leakage and inflammation in the same cohort of mice to understand how these measures relate to SAA changes in blood. All mice in this cohort were female. We found that mice exposed to 1 ppm O_3_ did not show statistically significant increases in weight loss vs. air control, whereas mice exposed to 3 ppm O_3_ in this cohort lost about 4.7% of their body weight, which was significantly different from the control and 1 ppm groups (Figure 2A). A dose of 1 ppm O_3_ caused no significant increase in serum SAA, with a mean concentration approximating that of air control. In contrast, 3 ppm O_3_ increased the serum SAA concentration by several orders of magnitude, which is consistent with what we have observed previously (Figure 2B) [25]. Both 1 ppm and 3 ppm O_3_ significantly increased the bronchoalveolar lavage (BAL) total protein concentration, a marker of altered alveolar capillary barrier function [42], to similar levels that were not statistically different from each other (Figure 2C). A dose of 3 ppm O_3_ significantly increased the BAL total cells (Figure 2D) and BAL neutrophils (Figure 2F) vs. control, whereas 1 ppm O_3_ did not. The BAL macrophage counts were not significantly different among groups (Figure 2E). A summary of the effects of the O_3_ doses in Balb/c mice is presented in Table 1.

**Figure 3:**
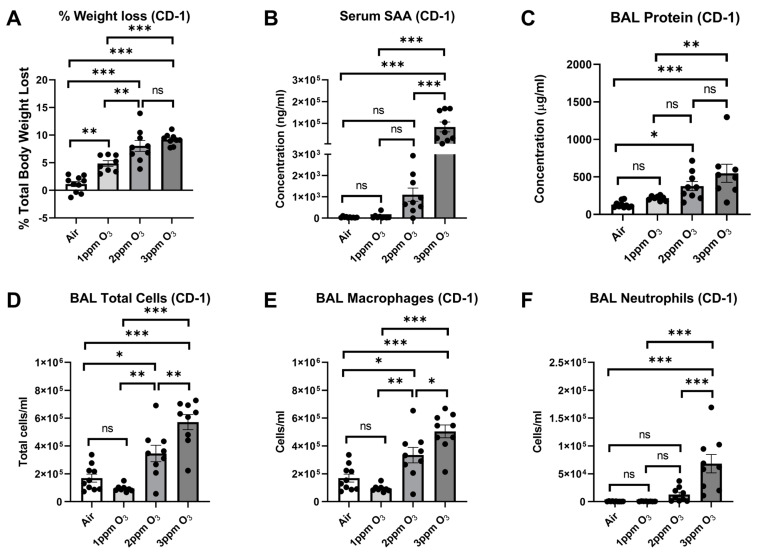
Effects of dose on O_3_-induced changes of weight loss (**A**), serum SAA (**B**), BAL total protein (**C**), and cellular markers of lung inflammation (**D**–**F**) in female CD-1 mice. All mice were studied 24 h post-exposure. The means ± SEM are graphed. N = 6–10/group, * *p* < 0.05, ** *p* < 0.01, *** *p* < 0.001 vs. indicated groups, ns = not significant (*p* > 0.1 except when reported).

Because of the apparent differences in responses to the 1 ppm and 3 ppm doses in female Balb/c mice, we next determined whether there was a similar dose-effect relationship of measured parameters in female CD-1 mice. CD-1 mice were chosen because we wanted to replicate our experiments in an outbred strain as a more rigorous model. We also included an intermediate dose of 2 ppm to have a more comprehensive understanding of the dose effects. Our findings in CD-1 mice supported a significant relation of O_3_ dose to the magnitude of changes in outcomes. Significant (*p* < 0.001) linear trends were noted for weight lost (F(1,33) = 25.62), total cells (F(1,32) = 56.28), total neutrophils (F(1,32) = 32.02), total macrophages (F(1,32) = 48.74), BAL protein concentration (F(1,31) = 24.51), and serum SAA (F(1,33) = 25.62), and differences in group means are shown in Figure 3 and summarized in Table 2.

### 2.2. Time-Response Effects of O_3_ on Body Weight, Serum SAA, and Pulmonary Damage and Inflammation

**Figure 4:**
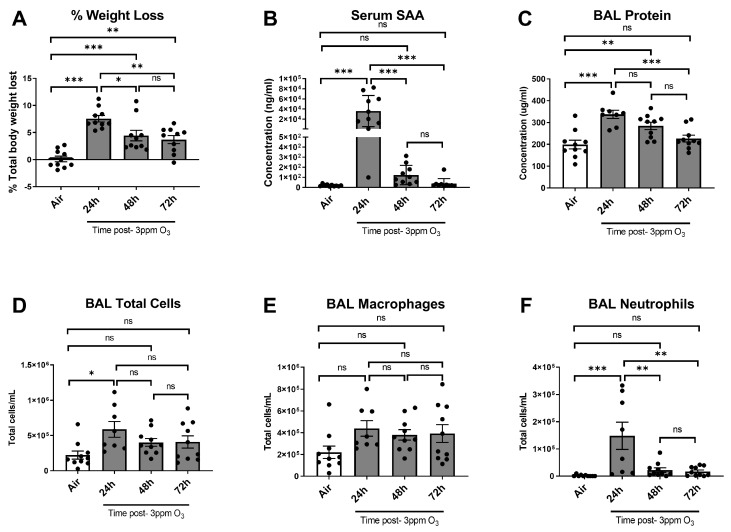
Effects of time after 3 ppm O_3_ exposure on weight loss (**A**), serum SAA (**B**), BAL total protein (**C**), and cellular markers of acute pulmonary inflammation (**D**–**F**) in female Balb/c mice. The air group was studied 72 h post-exposure. The means ± SEM are graphed. N = 8–10/group, * *p* < 0.05, ** *p* < 0.01, *** *p* < 0.001, ns = not significant (*p* > 0.1 except when reported) vs. indicated groups.

We next evaluated how long serum SAA increases persisted and associated changes in weight loss and pulmonary inflammation over time after a 3 ppm O_3_ exposure. In this cohort, all mice were female Balb/c and were exposed to O_3_ or air at the same time to ensure uniformity of the exposures. All mice were compared to a 72-h air control group to minimize the number of animals used. Weight loss after 24 h was slightly higher in this cohort vs. dose-response studies, with O_3_-exposed mice losing 7.6% of their initial body weight after 24 h, which was significantly different vs. air controls. Mice gained back some of their lost weight after 48 and 72 h, and the net weight loss was significantly lower at these time points vs. 24 h but remained significantly higher than air control (Figure 4A). The SAA levels in serum were significantly elevated at 24 h to similar concentrations observed in the dose-response cohort. The SAA concentrations were significantly reduced in blood by 48 h post-exposure, and although the mean was still arithmetically higher than the air control, there was not a statistically significant difference. The Serum SAA concentrations returned to air control levels by 72 h post-exposure (Figure 4B). Significant elevations in BAL total protein were observed up to 48 h post-exposure (Figure 4C), whereas significant elevations in BAL total cells and neutrophils were only significantly increased vs. air control at 24 h (Figure 4D,F). BAL macrophages showed an approximate doubling in numbers at all time points vs. air controls, but the increases were not statistically significant vs. air (Figure 4E). 

### 2.3. Effects of Sex on O_3_-Induced Changes in Body Weight, Serum SAA, and Pulmonary Damage and Inflammation

**Figure 5:**
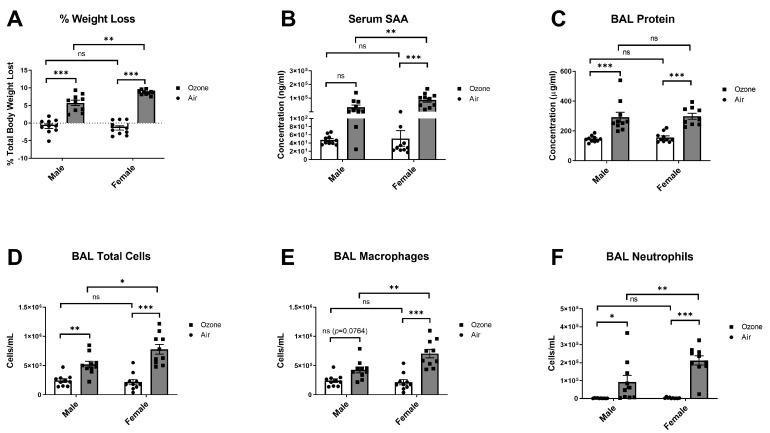
Effects of sex on weight loss (**A**), serum SAA (**B**), BAL total protein (**C**), and cellular markers of acute pulmonary inflammation (**D**–**F**) following 3 ppm O_3_ exposure in Balb/c mice. All groups were studied 24 h post-exposure. The means ± SEM are graphed. N = 9–10/group, * *p* < 0.05, ** *p* < 0.01, *** *p* < 0.001 vs. groups indicated, ns = not significant (*p* > 0.1 except when reported).

Sex differences in pulmonary responses to acute ozone exposures have been reported, with young females generally showing heightened inflammatory responses in the lungs and males showing greater airway hyperresponsiveness [43,44]. Considering that other physiological responses to O_3_ may vary by sex, we determined whether sex influenced SAA responses and associated changes in weight loss and pulmonary inflammation 24 h following a 3-ppm exposure to O_3_ in Balb/c mice. There was a significant interaction between the effects of sex and treatment on body weight loss (F(1,36) = 8.915, *p* = 0.0051), and main effects of sex and treatment on body weight loss were also significant (F(1,36) = 4.464, *p* = 0.0416 and F(1,36) = 201.3, *p* < 0.0001, respectively). The average weight of male mice prior to O_3_ exposure was 24.07 ± 1.23 g, and the average weight of females was 18.95 ± 1.15 g. Multiple comparisons testing showed significant differences in means of males and females exposed to air vs. O_3_ and a significant difference in mean % body weight lost in O_3_-exposed males vs females, with females showing more weight loss than males (Figure 5A). There was a significant interaction between the effects of sex and treatment on SAA levels in serum (F(1,36) = 5.784, *p* = 0.0214), and main effects of sex and treatment on serum SAA were also significant (F(1,36) = 5.785, *p* = 0.0214 and F(1,36) = 30.78, *p* < 0.0001, respectively). Multiple comparisons testing showed significant differences in means of females exposed to air vs. O_3_ and a significant difference in serum SAA levels in O_3_-exposed males vs. females, with females showing higher levels of serum SAA post-O_3_ exposure (Figure 5B). There was no significant interaction or main effect of sex on total BAL protein, but there was a significant main effect of treatment (F(1,33) = 50.59, *p* < 0.0001). Multiple comparisons testing showed significant differences in means of males and females exposed to air vs. O_3_, but no significant difference in means for O_3_-exposed males vs. females (Figure 5C). There was a significant interaction between the effects of sex and treatment on total BAL cells (F(1,36) = 6.088, *p* = 0.0185), and main effects of treatment were also significant (F(1,36) = 52.87, *p* < 0.0001). Multiple comparisons testing showed significant differences in means of males and females exposed to air vs. ozone and a significant difference in total BAL cells in O_3_-exposed males vs. females, with females showing a larger increase vs. males (Figure 5D). A significant interaction was observed between the effects of sex and treatment on BAL macrophages (F(1,36) = 8.587, *p* = 0.0058), and main effects of sex and treatment were also significant (F(1,36) = 5.779, *p* = 0.0215 and F(1,36) = 41.86, *p* < 0.0001, respectively). Multiple comparisons testing showed significant differences in means of females exposed to air vs. ozone and a significant difference in mean BAL macrophages in O_3_-exposed males vs. females, with females showing greater increases in BAL macrophages vs. males (Figure 5E). A significant interaction was observed between the effects of sex and treatment on BAL neutrophils (F(1,36) = 7.004, *p* = 0.0120), and main effects of sex and treatment were also significant (F(1,36) = 7.372, *p* = 0.0101 and F(1,36) = 45.22, *p* < 0.0001, respectively). Multiple comparisons testing showed significant differences in means of males and females exposed to air vs. O_3_ and a significant difference in mean BAL neutrophils in O_3_-exposed males vs females, with females showing greater increases in BAL neutrophils vs. males (Figure 5E). These observed sex differences are summarized in Table 1.

### 2.4. Dose-, Time-, and Sex-Dependent Effects of O_3_ on Sickness and Depressive-like Behaviors

**Figure 6:**
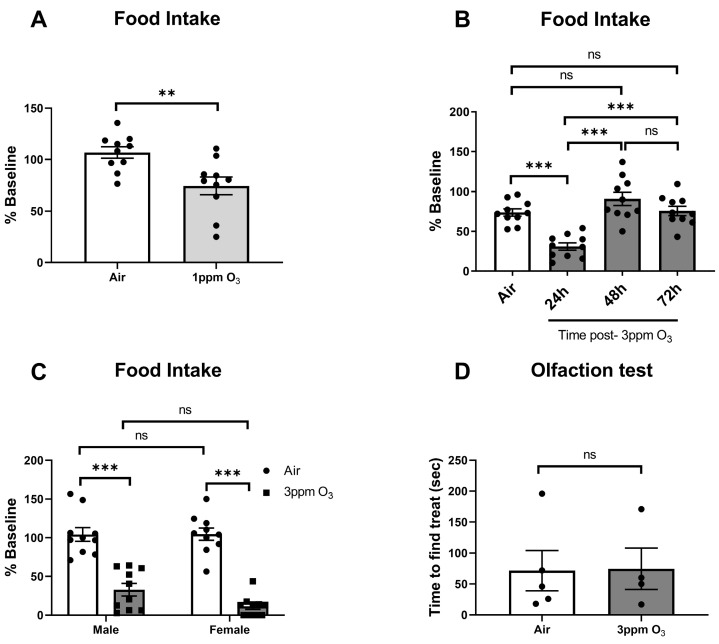
Effects of dose (**A**–**C**), time (**B**), and sex (**C**) on O_3_-induced changes in food intake, and effects of 3 ppm O_3_ on olfaction in female Balb/c mice (**D**). Except for B, all groups were studied 24 h post-exposure. The means ± SEM are graphed. N = 10/group (**A**–**C**), ** *p* < 0.01, *** *p* < 0.001 vs. groups indicated, ns = not significant (*p* > 0.1 except when reported).

**Figure 7:**
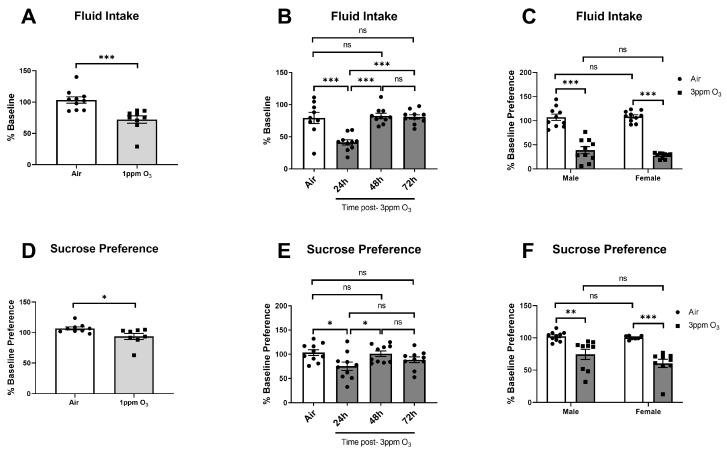
Effects of dose, time, and sex on O_3_-induced changes in total fluid intake (**A**–**C**) and sucrose preference in Balb/c mice (**D**–**F**). Except for **B** and **E**, all groups were studied 24 h post-exposure. The means ± SEM are graphed. N = 7–10/group, * *p* < 0.05, ** *p* < 0.01, *** *p* < 0.001 vs. groups indicated, ns = not significant (*p* > 0.1 except when reported).

**Figure 8:**
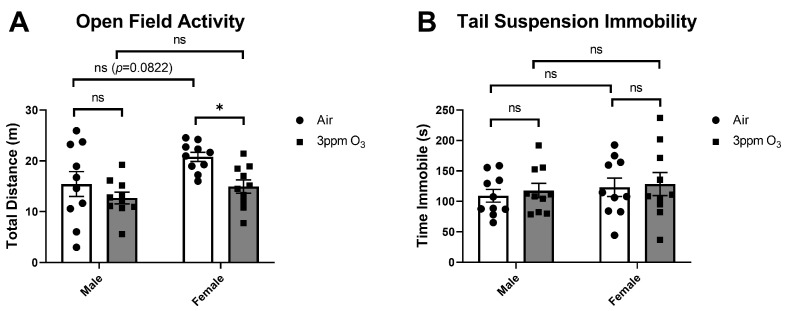
Effects of 3 ppm O_3_ on open field activity (**A**) and immobility on the tail suspension test in Balb/c mice (**B**). All groups were studied 24 h post-exposure. The means ± SEM are graphed. N = 9–10/group, * *p* < 0.05 vs. indicated groups, ns = not significant (*p* > 0.1 except when reported).

Acute inflammatory insults can cause behavioral changes that include reduced food and fluid intake, weight loss, reduced locomotor activity, anhedonia, and other behavioral sequelae that have many similarities with depression [38]. We first evaluated dose-, time-, and sex-dependent changes in food intake among mice exposed to O_3_. The doses and time points used were chosen to match the time points that were used to measure SAA and pulmonary leakage/inflammation, which was maximal at 24 h. We found that a 1 ppm O_3_ exposure caused about a 25% reduction in food intake vs. baseline at 24 h post-exposure in female Balb/c mice (Figure 6A). A 3 ppm O_3_ exposure caused a larger decrease in food intake in Balb/c female mice of about 70% (Figure 6B) to 90% (Figure 6C). Decreases in food intake in female mice persisted for 24 h and returned to baseline levels within 48 h (Figure 6B). In the male-to-female comparison, there was a main effect of treatment on food intake (F(1,36) = 119.5, *p* < 0.0001) but no significant effect of sex. Multiple comparisons testing also revealed a significant difference between air and O_3_ exposure within sexes, but food intake in male and female O_3_-exposed mice was not significantly different (Figure 6C). Prior to evaluating sucrose preference, we addressed the possibility that O_3_ may damage olfactory epithelium, causing olfactory dysfunction, which could confound behavioral tests that rely on the sense of smell or taste. We did not specifically investigate whether taste perception is altered, because tests used to evaluate taste perception are likely to be confounded by changes in taste preference that are known to occur as a component of sickness and depressive-like behavioral responses to inflammatory stimuli (78). Further, mice are obligate nasal breathers, so the majority of inhaled O_3_ would be encountered in the upper airway vs. the mouth. Our data show that the ability to locate a buried hidden treat was not altered in female mice exposed to 3 ppm O_3_ for 4 h vs. air-exposed mice (Figure 6D), indicating that a mouse’s ability to detect sucrose in their drinking solution would not be influenced by severe olfaction deficits in O_3_-exposed mice. We next administered the sucrose preference test in mice and reported both total fluid intake and % of baseline preference for sucrose; the baseline sucrose preference was about 80% in both sexes. Total fluid intake was acutely decreased by about 28% in 1 ppm O_3_-exposed mice 24 h after exposure, which was significantly different vs. control (Figure 7A). A dose of 3 ppm O_3_ induced more substantial reductions in total fluid intake of about 59% (Figure 7B) to 61% (Figure 7C) 24 h after exposure, which were also significantly different from air controls. The effects of O_3_ on fluid intake returned to the baseline by 48 h post-exposure (Figure 7B). Fluid intake in the male-to-female comparison showed a significant main effect of treatment on fluid intake (F(1,36) = 196.7, *p* < 0.0001), but there was no significant effect of sex or interaction between sex and treatment. We evaluated sucrose preference in the same cohort of mice by determining the change in sucrose preference from the baseline, which was measured the night prior to O_3_ exposure. Sucrose preference significantly decreased by about 6.2% in 1 ppm O_3_-exposed female mice 24 h after exposure (Figure 7D). A dose of 3 ppm O_3_ induced more substantial reductions in sucrose preference of about 24.7% (Figure 7E) to 39.6% (Figure 7F) in female mice 24 h after exposure which were significantly different from air control. The effects of O_3_ on sucrose preference returned to the baseline by 48 h post-exposure (Figure 7E). Sucrose preference in the male-to-female comparison showed a main effect of treatment on fluid intake (F(1,31) = 38.94, *p* < 0.0001), but there was no significant effect of sex or interaction between sex and treatment. A dose of 3 ppm O_3_ induced significant reductions in locomotor activity in female but not male mice (Figure 8A). Open field activity in the male-to-female comparison showed a significant main effect of treatment (F(1,36) = 7.485, *p* = 0.0096) and sex (F(1,36) = 5.836, *p* = 0.0209), but there was no significant interaction between sex and treatment. Neither O_3_ treatment nor sex had significant effects on tail suspension immobility time (Figure 8B). 

**Figure 9:**
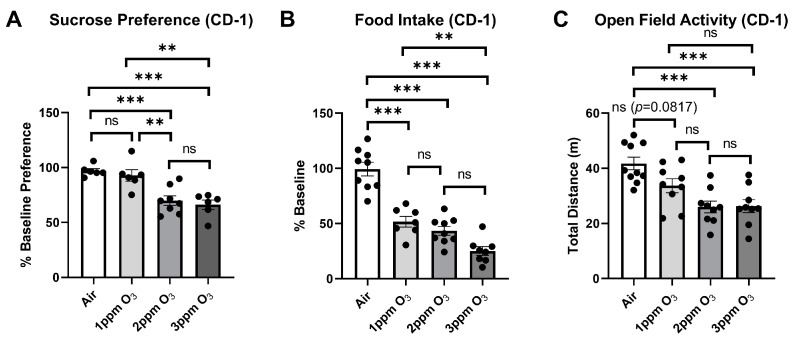
Effects of dose on O_3_-induced changes in food intake (**A**), sucrose preference (**B**), and Open Field Activity (**C**). The means ± SEM are graphed. N = 6–10/group, ** *p* < 0.01, *** *p* < 0.001 vs. indicated groups, ns = not significant (*p* > 0.1 except when reported).

In repeating the evaluation of sickness and depressive-like behaviors in female CD-1 mice following three different O_3_ doses, it was found that there was a significant (*p* < 0.001) linear trend relating O_3_ dose to measured parameters, which included food intake (F(1,29) = 119.5), sucrose preference (F(1,22) = 36.10), and open field activity (F(1,33) = 28.03). Group mean differences were also assessed, and significant differences are shown in Figure 9 and summarized in Table 2.

### 2.5. Biochemical Changes in the Kynurenine Pathway

**Figure 10:**
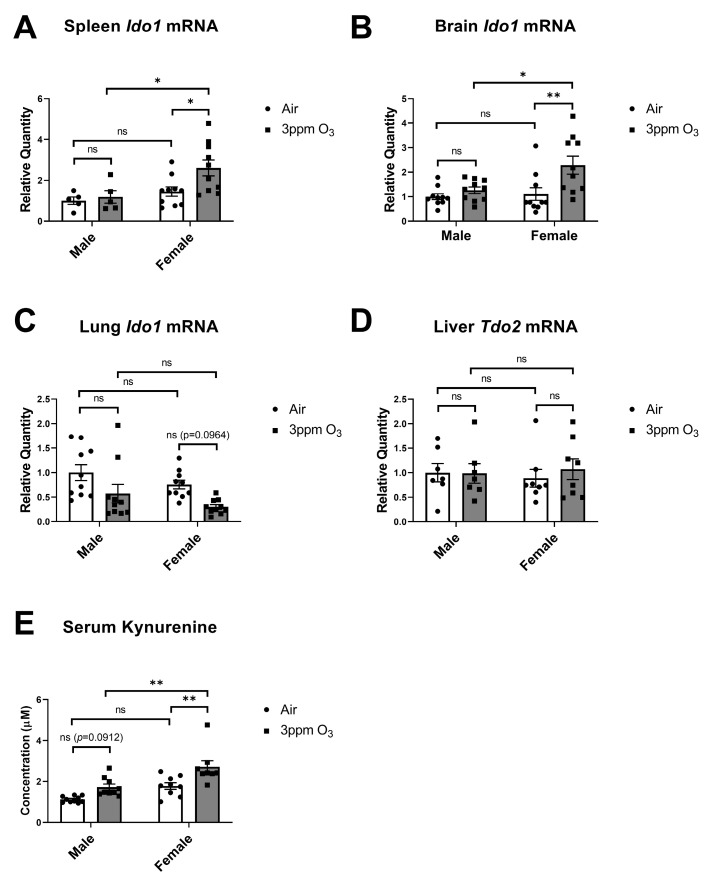
Effects of 3 ppm O_3_ on *Ido1* mRNA expression in spleen, brain, and lung (**A**–**C**), Tdo2 mRNA expression in liver (**D**), and kynurenine concentrations in serum in Balb/c mice (**E**). All groups were studied 24 h post-exposure. The means ± SEM are graphed. N = 8–10/group, * *p* < 0.05, ** *p* < 0.01 vs. indicated groups, ns = not significant (*p* > 0.1 except when reported).

IDO-dependent increases in circulating kynurenine have been found to mediate depressive-like behaviors but not sickness behaviors following an acute inflammatory stimulus [29,30]. Kynurenine is a product of tryptophan metabolism via two rate-limiting enzymes: IDO and tryptophan 2,3-dioxygenase (TDO). TDO is predominantly and constitutively expressed in the liver by the *Tdo2* gene [45]. IDO is expressed predominantly in extrahepatic tissues including epididymis, intestines, spleen, lung, and brain by the *Ido1* gene [46] and predominantly contributes to circulating kynurenine levels at the baseline and during inflammatory conditions [29,47], although TDO can also contribute to circulating kynurenine pools [48]. We therefore probed for *Ido1* mRNA expression in the spleen, brain, and lung (Figure 10A–C) and for *Tdo2* expression in the liver (Figure 10D). There was a significant main effect of sex (F(1,26) = 7.317, *p* = 0.0119) on *Ido1* expression in the spleen, and multiple comparisons testing showed that *Ido1* was significantly upregulated in the spleens of female mice. There was a significant main effect of sex and treatment (F(1,36) = 5.448, *p* = 0.0253, F(1,36) = 8.822, *p* = 0.0053, respectively) on *Ido1* expression in the brain, and multiple comparisons testing showed that *Ido1* was significantly upregulated in the brains of female mice. In contrast, the lungs showed a significant main effect of treatment (F(1,36) = 10.83, *p* = 0.0022) and were significantly downregulated in female mice. There were no significant effects of sex or O_3_ exposure on *Tdo2* expression. There was a significant main effect of sex and treatment on serum kynurenine levels (F(1,32) = 20.93, *p* < 0.0001, F(1,32) = 18.38, *p* = 0.0002, respectively). Multiple comparisons testing revealed that O_3_ induced significant kynurenine elevations in females but not males, which is consistent with the *Ido1* expression results. The results for the measured changes 24 h after exposure are summarized in Table 1.

### 2.6. Effects of O_3_ on SAA Triple Knockout (TKO) Mice

**Figure 11:**
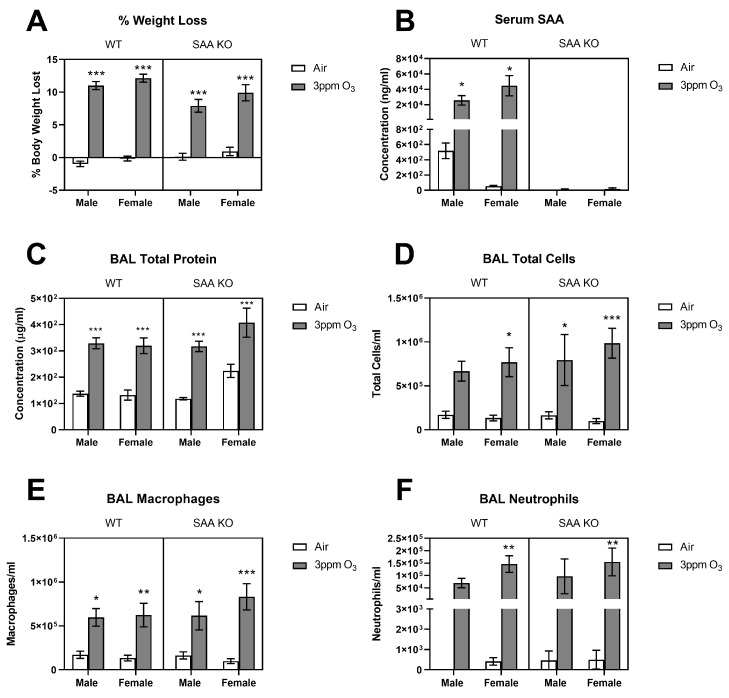
Effects of SAA1.1, 2.1, and 3 triple knockout on weight loss (**A**), serum SAA (**B**), BAL total protein (**C**), and cellular markers of acute pulmonary inflammation (**D**–**F**) 24 h after a 3 ppm O_3_ exposure. WT mice are background-matched C57BL/6J of the same age. The means ± SEM are graphed. N = 5–7/group, * *p* < 0.05, ** *p* < 0.01, *** *p* < 0.001 vs. air within sex and genotype.

**Figure 12:**
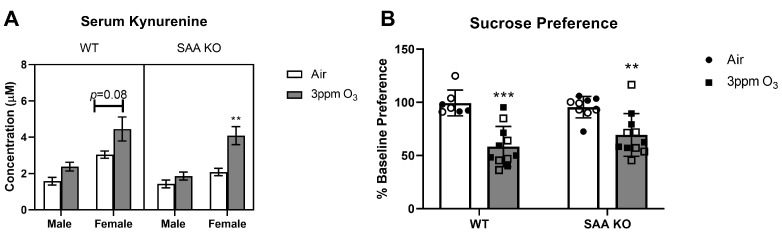
Effects of SAA1, 2, and 3 triple knockout on kynurenine concentrations in serum (**A**) and sucrose preference (**B**). In B, the filled shapes are male mice and unfilled are female mice. WT mice are background-matched C57BL/6J of the same age. The means ± SEM are graphed. N = 7–11 per group, ** *p* < 0.01, *** *p* < 0.001 vs. air within sex and/or genotype.

SAA has been reported to have pro-inflammatory activities in the lungs and can mediate depressive-like behavior in mice when overexpressed chronically [33,34,35,36,49]. Therefore, we next determined whether pulmonary inflammation, serum kynurenine, and behavioral responses to 3 ppm O_3_ differed in mice lacking all three inflammatory isoforms of *Saa* (*Saa*1.1, 2.1, and 3 triple knockout, SAA TKO) vs. their wild-type (WT) C57BL/6J controls. We found that the % change in body weight significantly varied by sex (F(1,42) = 5.577, *p* = 0.0229) and exposure type (F(1,42) = 413.2, *p* < 0.0001), and there was a significant genotype x exposure interaction (F(1,42) = 13.75, *p* = 0.0006). The mean % weight loss post-O_3_ was slightly lower for SAA TKO mice of both sexes vs. WT (Figure 11A), supporting that SAA contributed slightly to O_3_-induced weight loss. As expected, SAA was induced by O_3_ in serum of WT but not SAA TKO mice, verifying the genotype (Figure 11B). O_3_ significantly increased BAL protein and macrophages in males and females of both WT and SAA TKO mice (Figure 11C,E) and significantly elevated neutrophils in females of both WT and SAA TKO mice (Figure 11F). The BAL total cells were significantly increased in all groups by O_3_ except WT males (Figure 11D). A three-way ANOVA analysis only showed significant main effects of exposure for all BAL cell measures. There was a significant main effect of sex (F(1,43) = 5.86, *p* = 0.0197) and exposure (F(1,43) = 102.7, *p* < 0.0001) and a significant interaction of sex x genotype F(1,43) = 7.880, *p* = 0.0075) on the total BAL protein levels. However, there were no significant genotype x exposure effects for any group, indicating that SAA did not contribute to O_3_-induced pulmonary damage or inflammation. The serum levels of kynurenine were significantly elevated by O_3_ only in the SAA TKO female group, although there was a statistical trend of an increase in WT females (Figure 12A). There was a significant main effect of sex (F(1,32) = 42.41, *p* < 0.0001) and exposure (F(1,32) = 22.35, *p* < 0.0001) as well as a sex x exposure interaction (F(1,32) = 4.953, *p* = 0.0332), indicating that kynurenine is selectively elevated in female C57BL/6J mice, which is similar to our findings in the Balb/c strain. However, SAA did not contribute to ozone’s effects on circulating kynurenine. Finally, we determined whether SAA affected sucrose preference. Since there was not an apparent sex difference in sucrose preference post-ozone, we combined approximately equal numbers of each sex to increase statistical power to detect genotype effects. While sucrose preference was reduced in both WT and SAA TKO groups post- O_3_ exposure (Figure 12B), there was no significant genotype effect, showing that SAA does not mediate this O_3_-induced depressive-like behavior.

## 3. Discussion

Although O_3_ primarily targets the lungs, O_3_ exposure can also have effects on distal organs that include the CNS [8,50,51]. How O_3_ alters CNS functions is incompletely understood, but recent works support the concept of a lung-brain axis mechanism, whereby circulating factors are upregulated in response to pulmonary inflammation [12,25]. These circulating factors can then exert their effects in the brain directly if they can cross the BBB or may have indirect effects by modifying functions of brain barriers or activating regions of the brain that lack a BBB such as circumventricular organs. Two circulating factors that are induced by O_3_ and can cross the intact BBB are SAA and kynurenine. Both molecules have neuromodulatory functions, have been associated with human depression, and can cause depressive-like behaviors in rodents [29,30,35,36]. To better understand the relations of pulmonary inflammation, neurobehavioral changes, and circulating SAA/kynurenine, we conducted dose-response and time-response studies following a 4-h single exposure to O_3_ and determined whether these responses varied by sex. We further evaluated whether mice lacking all three inflammation-induced SAA isoforms varied in their responses to ozone vs. their wild-type counterparts.

Our prior work in mice showed that O_3_ increases production of SAA in the liver, leading to increased SAA in blood which then crosses the intact BBB [25]. We found that SAA increases in blood were strongly and positively correlated with cellular markers of pulmonary inflammation [25]. However, this previous study only investigated responses to O_3_ 6 and 24 h following a 3-ppm dose, which elicits a high level of pulmonary inflammation in mice relative to that observed in O_3_-exposed humans [6,7,52,53]. To better understand the relations of SAA induction, pulmonary inflammation, and behavioral responses to O_3_, we conducted dose-response studies at 1 ppm and 3 ppm in female Balb/c mice and 1, 2, and 3 ppm in female CD-1 mice. These studies allowed us to estimate the effective dose needed to elicit changes in systemic biomarkers, pulmonary inflammation, and behaviors, and whether these responses were consistent in commonly used mouse strains. Our time response studies aimed to further characterize the resolution of these endpoints. We showed that significant SAA elevations in blood occurred 24 h after a 3 ppm but not a 2 ppm or 1 ppm O_3_ exposure in both Balb/c and CD-1 mice. Increased BAL total cell counts and neutrophil counts were also significantly elevated only at the 3 ppm O_3_ dose, which supports a relation of hepatic SAA induction and release into blood with pulmonary neutrophilia. Statistically significant increases of BAL macrophages were not detected in Balb/c mice at any ozone dose used, although BAL macrophages were increased at 2 ppm and 3 ppm doses in CD-1 mice and at 3 ppm in C57BL/6J mice, suggesting different strain sensitivities for macrophage recruitment. The total BAL protein, which is a marker of alveolar-capillary barrier function [42], was equally elevated by 1 ppm and 3 ppm O_3_ in Balb/c mice and in a dose-dependent manner in CD-1 mice. The finding that BAL protein elevations occurred at a lower O_3_ dose that did not induce significant increases in SAA suggests that SAA does not contribute to alveolar-capillary barrier dysfunction in this model, and this was verified in SAA knockout mice. Food intake, fluid intake, and sucrose preference were also significantly reduced by both 1 ppm and 3 ppm O_3_ in Balb/c mice, but 3 ppm O_3_-induced decrements were greater in magnitude. There was a dose-dependent relation of these parameters in CD-1 mice. These findings indicate that there is a dose-effect of O_3_ on sickness and some depressive-like behaviors that occur even in the absence of detectable increases in SAA and immune cell trafficking to the lungs.

Time-response studies showed that O_3_’s effects on SAA levels in blood, markers of lung damage and inflammation, and measures of sickness and depressive-like behaviors were most substantial at 24 h post-exposure, and most of the measured parameters returned to control levels by 48 h post-exposure. The parameters affected by O_3_ that remained changed at 48–72 h included body weights and total BAL protein. Elevations in BAL protein persisted up to 48 h but then returned to normal by 72 h. The persistence of increased BAL protein up to 48 h post-exposure is consistent with time-course findings reported previously [54] and indicates that disruption of the alveolar-capillary barrier persists even after inflammatory markers measured here have returned to baseline. Mice lost a significant amount of weight 24 h post O_3_-exposure at 3 ppm, which was partially regained by the 48- and 72-h time points. Food intake and fluid intake also returned to normal by 48 h post-exposure, but there was no compensatory increase above the baseline, which might explain why weights did not return to baseline levels by 72 h. Thus, resolution of behavioral effects of O_3_ occurred together with resolution of SAA and pulmonary inflammation. 

Although prior work has supported that SAA is a mediator of depressive-like behaviors and lung neutrophilia [35,49,55], we found that SAA does not contribute to O_3_-induced lung inflammation or vascular leakage, and does not have a significant effect on sucrose preference, a measure of depressive-like behavior. Additionally, O_3_ at concentrations that stimulated SAA did not alter performance on the tail suspension test, which further suggests that upregulation of endogenous SAA does not universally contribute to depressive-like behaviors or pulmonary inflammation. Rather, its activities may be disease- or model-specific. We did find that SAA moderately contributes to O_3_-induced weight loss, although our findings that weight loss also occurred in the absence of SAA upregulation at lower O_3_ doses indicate that it is not the only mediator. Similar to findings with LPS models, there may be distinct mechanistic underpinnings that contribute to sickness vs. depressive-like behaviors post-ozone [29,56]. 

One limitation of our findings is that the 3 ppm O_3_ dose elicits more severe pulmonary inflammation in mice than what has been reported in humans exposed to relatively high experimental concentrations of O_3_ [52,53]. Increased levels of lung neutrophils are noted in humans following O_3_ exposures in the 0.2–0.25 ppm range [57,58], but it is unclear whether SAA levels in blood correlate with lung neutrophils in humans as they do in mice following O_3_ exposure. Formal studies are also lacking on transient neurobehavioral symptoms post-O_3_. The relation of SAA induction in blood and lung neutrophilia is consistent with prior works from other groups who have showed that SAA may be a systemic marker of pulmonary injury. For example, it has been shown that SAA levels in lungs of chronic obstructive pulmonary disease (COPD) patients positively correlate with elastase-positive neutrophils, and that increases in circulating SAA can predict severity of acute exacerbations of COPD [34]. Interestingly, chronic intranasal treatments of recombinant SAA induced neutrophilic airway inflammation [49], although it has been shown that recombinant and endogenous/lipid-bound SAA have distinct biological activities [59]. Mouse SAA3 was also shown previously to be a mediator of IL-6-dependent pulmonary inflammation through its ability to induce IL-17A [60]. Our findings are consistent with another recent study showing that SAA TKO does not affect adipose inflammation in a mouse obesity model, but significant effects on glucose metabolism were noted, with obese SAA TKO mice performing worse on intraperitoneal glucose tolerance tests vs. their wild-type counterparts [61]. Although our work shows that SAA does not contribute to O_3_-induced lung inflammation, future studies are needed to determine whether O_3_-induced lung inflammation drives hepatic SAA production or whether the two features are driven by separate mechanistic underpinnings.

Sex differences were observed for many measured parameters following O_3_ exposure, including the magnitude of weight loss, pulmonary inflammation, and circulating levels of SAA. Our findings are consistent with other studies showing that female mice have exacerbated pulmonary inflammation in response to O_3_ [43,44]. However, we were not able to collect data on dosimetry, and so we cannot rule out sex differences in O_3_ deposition due to size disparity. A prior study that evaluated of O_3_ deposition in adults vs. pups showed that there was less overall O_3_ deposition in the pups, suggesting that smaller size reduces O_3_ deposition [62]. Therefore, we would expect a size disparity to favor slightly more O_3_ deposition in male mice. Increases in circulating kynurenine and in *Ido1* mRNA levels in brain and spleen were specifically apparent in female mice, although male-female comparisons post-O_3_ were not significantly different and there was not an interaction between sex and treatment. In lungs, *Ido1* expression was arithmetically decreased in both sexes, although the difference was only statistically significant in females. The reduction of *Ido1* in lungs is consistent with increased lung neutrophilia that occurs with O_3_ since IDO negatively regulates neutrophil trafficking [63]. Levels of liver *Tdo2* were unchanged, suggesting that *Ido1* could be a predominant mediator of the systemic increases in kynurenine. However, future work is needed to verify that increases in circulating kynurenine are IDO-dependent and to identify the prevailing tissue that contributes to increases in circulating kynurenine. These findings also implicate the kynurenine pathway as a second systemic mediator in the lung-brain axis whose expression is independent of SAA. Kynurenine can cross the intact blood-brain barrier via the large neutral amino acid transporter [32], and the majority of brain kynurenine is derived from blood under physiological conditions. In inflammatory states, nearly all kynurenine in the brain comes from blood [64]. In the brain, kynurenine can be metabolized to neuroprotective or neurotoxic mediators in a cell-type dependent manner. In the healthy brain, kynurenine is predominantly metabolized by astrocytes, which produce kynurenic acid, which is neuroprotective at physiological levels [65]. Microglia express enzymes that metabolize kynurenine into neurotoxic products such as the radical-generating 3-hydroxykynurenine and quinolinic acid, which can cause excitotoxic cell death [30]. Under inflammatory conditions, kynurenine, 3-hydroxykynurenine, and quinolinic acid are increased in the brain [66] and these increases have been proposed to contribute to neurological diseases like Alzheimer’s and depression [31]. Our findings corroborate recent studies in rats, which showed that acute O_3_ exposure increases circulating levels of kynurenine, and in this model, kynurenine upregulation was not significantly affected by the glucocorticoid inhibitor metyrapone [26]. In another study that investigated the effects of maternal O_3_ exposure and/or high-fat diets on the metabolomes of male and female offspring in rats, it was found that maternal exposure to O_3_ significantly increased the circulating kynurenine concentrations in female but not male offspring. Male offspring kynurenine levels were arithmetically higher than their female counterparts born from air or O_3_-exposed dams but unaltered by maternal O_3_ exposure [67]. A limitation of our work is that we did not quantify kynurenine and its metabolites in the brain, and so, future work is needed to evaluate how systemic elevations in kynurenine might affect CNS kynurenine metabolites and their functions in context of O_3_ exposures. Our prior work has shown that 3 ppm O_3_ does not cause increases in brain or blood cytokines [25], suggesting that the inflammatory links of O_3_ to CNS dysfunction do not involve classical pro-inflammatory cytokine-mediated responses such as those to pathogen-associated stimuli. 

In conclusion, our work shows that O_3_ exposure has dose-dependent effects on sickness and depressive-like behavior responses, which are detectable in the presence and absence of overt pulmonary inflammation and systemic increases of SAA. Female mice have stronger upregulation of SAA, kynurenine, pulmonary inflammation vs. male mice, and worse open field activity in response to O_3_. SAA contributes to weight loss at higher ozone doses without affecting pulmonary inflammation or injury and systemic kynurenine, although its strong correlation with neutrophils suggests some utility as a biomarker. Future work is needed to elucidate the mechanisms of neurobehavioral responses to O_3_ and the extent to which they impact brain health.

## 4. Materials and Methods

### 4.1. Vertebrate Animals

All mice were treated in accordance with NIH Guidelines for the Care and Use of Laboratory Animals in an AAALAC-accredited facility and approved by the Institutional Animal Care and Use Committee of the VA Puget Sound Health Care System (VAPSHCS). Male and female BALB/c mice and female CD-1 mice were purchased from Charles River (Wilmington, MA, USA), allowed to adapt for 1–2 weeks following shipment and were studied at 10–12 weeks of age. *Saa*1/2/3 triple knockout (SAA TKO) mice were a gift from Dr. Nancy Webb at the University of Kentucky and were originally generated by Drs. June-Yong Lee and Dan R. Littman by the Rodent Genetic Engineering Core (RGEC) at NYULMC [55]. The SAA TKO mice were maintained on a C57BL/6J background and bred in-house at the VAPSHCS to generate the mice used in this study. C57BL/6J WT control mice were also obtained from Dr. Nancy Webb and bred in-house. Mice were kept on a 12/12-h light/dark cycle (6:00–18:00 lights on) with ad libitum food and water, except during exposures to O_3_ when food was withheld. 

### 4.2. Ozone Exposure

Just prior to exposure, mice were group-housed (n = 3–4/cage) in standard mouse cages with wire tops that lack bedding. Individual mice were identified using an animal-safe marker. Food was withheld for the duration of exposure to prevent consumption of ozonated food, and water was provided ad libitum. Up to 4 cages at a time were placed in a 30″ × 20″ × 20″ polypropylene chamber, where O_3_ (3 ppm, 2 ppm, or 1 ppm, chamber 1) or compressed dry air (chamber 2) was pumped into the chamber at equivalent rates. Because we only had two chambers, only one O_3_ dose was co-exposed with air on a given day. However, O_3_ exposures at each dose were replicated at least once to capture day-to-day variability, and results for each dose showed consistent trends. Males and females were co-exposed in the same chamber for studies that compared sex. Each chamber is equipped with a small fan near the infusion site that ensures even dispersion of the infused gas throughout the chamber. The temperature was maintained at 21–24 °C and the humidity at 35–49% for both chambers. O_3_ levels in the chambers were generated and regulated using an Oxycycler AT42 system (BioSpherix, Parish, NY, USA). Prior to each experiment, the system was calibrated using a model 106-L O_3_ detector (2B Technologies, Boulder, CO, USA), and O_3_ levels were recorded from an inlet valve in one of the mouse cages every 10 s for the duration of exposures. In all studies, O_3_ achieved its target concentration within 10 min, and levels were regulated within 10% of the target concentration (1 ppm +/− 0.1 ppm and 3 ppm +/− 0.3 ppm) thereafter. All exposures were conducted for 4 h (10:00–14:00), and mice were then returned to their home cages. Figure 1 depicts the set-up of our O_3_ exposure paradigm with respect to timing of behavioral assays and tissue collection. Behavioral testing and tissue collection was done in a randomized order so that each group was evenly dispersed across each time window of testing to mitigate nuisance variables. 

Although O_3_ concentrations that are environmentally relevant to humans are much lower than those used in this study, it has been shown that higher concentrations of O_3_ are needed to elicit similar pulmonary responses in experimental exposures of rodents vs. humans. For example, healthy young men exposed to an environmentally relevant dose of 0.1 ppm O_3_ for 6.6 h with moderate exercise exhibited over a 350% increase in BAL neutrophils and over a 20% increase in BAL protein 18 h post-exposure [52]. Healthy young men exposed to 0.4 ppm O_3_ for 2 h with exercise had more robust responses, with neutrophils increasing over 8-fold and BAL protein increasing 2-fold [53]. In our study, female BALB/c mice exposed to 1 ppm O_3_ exhibited an 8.8-fold increase in BAL neutrophils (although this was not significantly different from controls), and a 0.5-fold increase in BAL protein. Therefore, the magnitude of biological responses observed at 1 ppm in mice are similar to those reported in human studies using a short-duration 0.4 ppm dose with intermittent exercise in healthy human males. This difference in humans and mice may be explained, in part, by less O_3_ deposition in the airway under typical rodent exposure conditions that do not involve exercise [68], as well as a greater resistance of rodents to O_3_-induced damage and inflammation [69]. The 3-ppm dose elicits higher fold-changes in BAL and neutrophils, but we included this dose for comparison to our prior work for the purpose of comparing to lower doses of 2 ppm and 1 ppm O_3_.

### 4.3. Sucrose Preference Test

A decreased preference for sucrose as measured by this test is taken as evidence for depression, and sucrose preference testing was carried out based on standard methodologies that do not withhold food [70]. Mice were single-housed and adapted to drinking water from two sipper bottles filled with water for 3 days. The morning after habituation, one of the water bottles was replaced with a 3% sucrose solution, which was determined to be the optimal concentration of sucrose to achieve about 80% preference on average for the Balb/c strain and 90% on average for the CD-1 strain. Overnight liquid consumption was then evaluated by weighing the bottles at 15:00 and again at 8:00. Baseline sucrose consumption was recorded for three nights, and each morning the bottles were switched in their left/right orientation. Sucrose preference testing post-ozone commenced at 15:00 the day of exposures and bottles were re-weighed at 8:00 the next day. Mice showing a baseline sucrose preference of 50% or lower (which we found occurs at about a 10% rate in Balb/c mice) were excluded from analysis. Mice were also occasionally excluded if there was evidence of leakage or drainage of either sipper bottle by the mouse.

### 4.4. Open Field Test

In this test, decreased locomotor activity is taken as evidence for sickness behavior [29]. At 8:30 the morning after O_3_ exposure, mice were placed in a quiet room with dim light (8–10 lux) and allowed to adapt for 2 h. They were then placed into the center of a square open field with dimensions 40 × 40 cm and activity in the field was recorded for 10 min using ANY-maze video tracking software version 6.35 (Stoelting Co. Wood Dale, IL, USA). The center of the field was defined as a square with dimensions 20 × 20 cm. The order of testing was randomized such that mice from each group were evenly distributed over the testing period.

### 4.5. Tail Suspension Test

In this test, an increase in the time spent immobile vs. control is taken as evidence for depressive-like behavior. Experiments were carried out according to established methods for this test [71]. Briefly, medical tape was placed around the mouse’s tail leaving some slack, and the slack of the tape was fastened using a binder clip. Thin, rigid plastic tubes were placed over the mouse tails to prevent tail climbing. The binder clip was hung on a hook, fastened to a 25 cm (w) × 40 cm (L) × 20 cm (D) plastic box with the open end facing towards a camera. An empty mouse cage with a foam pad covered by absorbent bench paper was placed under the mouse in the event of a fall. Within about 10 s of securing the mouse, movement was tracked for 6 min using ANY-maze video tracking software version 6.35 (Stoelting Co., Wood Dale, IL, USA). The order of testing was randomized such that mice from each group were evenly distributed over the testing period. 

### 4.6. Olfaction Test

As an impaired sense of smell can affect performance in the sucrose preference test, olfaction was tested in our mice using the buried food test [72]. Mice were habituated to Froot Loop ^®^ treats for three nights prior to study by providing about 5 per cage in the afternoon, and palatability of the treats was confirmed by verifying their consumption the next morning. Mice were then exposed to air or 3 ppm O_3_ from 10:00–14:00, returned to home cages with food from 14:00–17:30, and fasted overnight until olfaction testing the next morning at 8:00. The next morning, mice were placed in a cage with about 3 inches of bedding that contained a buried Froot Loop ^®^ treat in one of the corners. The mice were monitored and the time to find the treat was recorded. Due to the potential confounds of fasting, these mice were not included in assessments of other behavioral, physiological, or biochemical parameters.

### 4.7. Tissue Collection and Processing

Mice were anesthetized with 2 mg/g urethane, and blood was harvested from the abdominal aorta, allowed to clot at room temperature for 30 min, and then centrifuged at 2500× *g* for 10 min. Serum was collected, aliquoted and stored frozen. Brains were collected by severing the spinal cord, leaving the trachea intact for BAL, and then cut in half sagittally. The left medial lobes of the liver and spleen were also dissected and cut in half. One half of each tissue was snap-frozen in liquid nitrogen, and the other half was placed in RNA later and frozen. BAL was performed on mice by perfusing and aspirating the lungs 3 times with 1 mL sterile phosphate buffered saline. BAL fluid was stored on ice and centrifuged at 200× *g* for 5 min at 4 °C. The supernatant was removed, aliquoted, and frozen, and 0.5mls of supernatant was reserved to resuspend the cell pellet. Total cell counts were determined from the cell suspension by manual counting using a hemacytometer. Differential cell counts were performed on cytocentrifuge preparations (Cytospin 3, Thermo Fisher Scientific, Waltham, MA, USA) that were stained with Hemacolor (Sigma-Aldrich, St. Louis, MO, USA). Cell counts were performed using Image J version 1.52p, and at least 200 cells were counted to determine relative cell proportions. Exclusion of datapoints occasionally occurred if BAL was not recovered or if the cytospin slides were not countable.

### 4.8. SAA ELISA

SAA mouse duoset kits (R and D systems, Minneapolis, MN, USA) were used to quantify SAA in serum. Serum was thawed and diluted 1/50 for controls and 1/10,000 for ozone-exposed samples using diluent specified in the kit. ELISAs were carried out according to manufacturer instructions. Some samples are missing because there was not sufficient serum recovered from some of the mice in the cohort. 

### 4.9. Kynurenine ELISA

IDK high sensitive Kynurenine ELISAs (Immundiagnostik, Bensheim, Germany) were used to quantify kynurenine in serum. Serums and standards were diluted and derivatized, and ELISAs carried out according to kit instructions. Some samples are missing because there was not sufficient serum recovered from some of the mice in the cohort.

### 4.10. RNA Extraction and Ido1 Measurement

Tissues preserved in RNA later were thawed and homogenized in Qiazol. RNA was extracted from tissue homogenates using RNeasy Plus Universal Kits (Qiagen, Valencia, CA, USA). Superscript IV first strand kit (Thermo Fisher Scientific, Waltham, MA, USA) was used to convert 5 µg RNA template to cDNA in a 20 uL reaction volume. For qPCR, 120 ng cDNA was amplified using TaqMan Fast Advanced Master Mix (Thermo Fisher, Waltham, MA, USA). TaqMan primer/probe sets with FAM-MGB probe (Thermo Fisher, Waltham, MA, USA) were used to amplify cDNA and included mouse *Ido1* (Mm00492590_m1), *Tdo2* (Mm00451269_m1), and *Gapdh* (Mm99999915_g1). The delta-delta Ct method was used to calculate relative changes in *Ido1 and Tdo1* expression among treatment groups. Some samples are missing from spleen and liver mRNA measurements due to poor RNA quality following extraction. 

### 4.11. Statistical Analysis

Analysis of dose- and time- response data with three or more groups was carried out by one-way ANOVA and individual groups compared using Tukey’s multiple comparisons test. Linear trends were also evaluated. Analysis of two groups was carried out by two-tailed unpaired *t*-tests. Analysis of sex-dependent responses to O_3_ was done using two-way ANOVA and individual groups were compared using Sidak’s multiple comparisons test. Analysis of the effects of SAA knockout on O_3_ responses was done using three-way ANOVA for parameters where effects of sex were evaluated, and by two-way ANOVA when both sexes were analyzed in the same group. For ANOVA analysis, the F statistics are reported along with the degrees of freedom of the numerator and denominator (DFn, DFd). All data were analyzed using the Prism software package version 8.3.0 (GraphPad Inc, San Diego, CA, USA).

## Figures and Tables

**Table 1:** Summary of significant dose and sex-dependent changes induced by O_3_ in Balb/c mice.

		1 ppm O_3_ (Either Sex)	3 ppm O_3_ (Either Sex)	Male vs. Female Difference
Pulmonary Inflammation/Damage Markers	BAL protein	↑	↑	No
	Total BAL cells	N.S.	↑	Yes
	BAL macrophages	N.S.	N.S.	Yes
	BAL Neutrophils	N.S.	↑	Yes
Systemic Inflammatory markers	Serum SAA	N.S.	↑	Yes
	Serum kynurenine	N.D.	↑	No
	Brain *Ido1*	N.D.	↑	No
	Spleen *Ido1*	N.D.	↑	No
Fluid intake	Weight loss	N.S.	↑	Yes
	Food intake	↓	↓	No
	Fluid intake	↓	↓	No
	Open field activity	N.D.	↓	No
Open field activity	Sucrose preference	↓	↓	No
	Tail suspension immobility	N.D.	N.S.	No

↑ and ↓ indicate an increase or decrease vs. control, respectively. N.S. = no significant difference with O_3_, N.D. = not determined.

**Table 2:** Summary of significant dose-dependent changes induced by O_3_ in female CD-1 mice.

		1 ppm O_3_	2 ppm O_3_	3 ppm O_3_
Pulmonary Inflammation/Damage Markers	BAL protein	N.S.	↑	↑
	Total BAL cells	N.S.	↑ *	↑ *
	BAL macrophages	N.S.	↑ *	↑ *
	BAL Neutrophils	N.S.	N.S.	↑ *
Systemic Inflammatory markers	Serum SAA	N.S.	N.S.	↑ *
Sickness Behaviors	Weight loss	N.S.	↑ *	↑
	Food intake	↓	↓	↓
	Open field activity	N.S.	↓	↓
Depressive-like Behaviors	Sucrose preference	N.S.	↓ *	↓

↑ and ↓ indicate an increase or decrease vs. air, respectively. An * indicates that the change was significantly different from the next lowest dose (i.e., 3 ppm vs. 2 ppm or 2 ppm vs. 1 ppm). N.S. = no significant difference with O_3_, N.D. = not determined.

## Data Availability

All data are available upon reasonable request from the corresponding author.
